# A New High Hydrostatic Pressure Process to Assure the Microbial Safety of Human Milk While Preserving the Biological Activity of Its Main Components

**DOI:** 10.3389/fpubh.2018.00306

**Published:** 2018-11-06

**Authors:** Gérard Demazeau, Adrien Plumecocq, Philippe Lehours, Patrice Martin, Leslie Couëdelo, Claude Billeaud

**Affiliations:** ^1^HPBioTECH, Gradignan, France; ^2^Laboratoire de Bacteriologie, Centre Hospitalier Universitaire de Bordeaux, Bordeaux, France; ^3^UMR1313 GABI, INRA, AgroParisTech, Université Paris-Saclay, Jouy-en-Josas, France; ^4^Department Nutrition-Health & Lipid biochemistry of ITERG, Bordeaux, France; ^5^Neonatology Nutrition, Lactarium Bordeaux-Marmande, CIC Pédiatrique 1401 Children's Hospital, Bordeaux, France

**Keywords:** human milk, HHP, pasteurization, human milk bank, spores, lipase, immune proteins, CMV

## Abstract

**Background:** The main process used to pasteurize human milk is the low-temperature, long-time Holder method. More recently, the high-temperature, short-time method has been investigated. Both processes lead to the appropriate inactivation of vegetative bacterial forms but are ineffective against bacterial spores.

**Research Aims/Questions:** We aimed to accomplish two main objectives: inactivation of all pathogens, including spores; and preservation of the activity of milk components.

**Design/Methods:** Recently, a novel high-hydrostatic pressure process has been developed by HPBioTECH. Using the same raw human milk samples, we compared the effects of this method with those of the Holder method on vegetative and spore forms of pathogens and on bioactive components (lipase activity, immunoproteins).

**Results:** Two main microbial strains were selected: *Staphylococcus aureus* (as a reference for vegetative forms) and *Bacillus cereus* (as a reference for spores). Use of the high-hydrostatic pressure process led to microbial decontamination of 6 log for both *S. aureus* and *B. cereus*. Additionally, the bioactivity of the main components of human milk was preserved, with activities of lipase, α-lactalbumin, casein, lysozyme, lactoferrin, and sIgA of ~80, 96–99, 98–100, 95–100, 93–97, and 63–64%, respectively.

**Conclusions:** Use of this novel high-hydrostatic pressure process to generate microbiologically safe human milk may provide important benefits for preterm infants, including improved assimilation of human milk (leading increased weight gain) and improved resistance to infections. Because 10% of all human milk collected is contaminated by *B. cereus*, use of this method will also prevent waste.

## Introduction

Human milk is the appropriate standard nutrient for infant development (1) and is also given to preterm and very low birth weight infants (2, 3).

Two types of pathogens can contaminate such a medium: (i) endogenous pathogens from the mother and (ii) exogenous pathogens that mainly result from human milk collection by milk banks (4). Consequently, ensuring the safety and quality of donor human milk appears to be a crucial issue (5–8).

The main processes used for human milk pasteurization are based on thermal pathogen inactivation: (i) the low-temperature, long-time (LTLT) method (62.5°C; 30 min) (5, 6), which is also called the Holder method (traditionally developed in milk banks); and (ii) the high-temperature, short-time (HTST) method or flash heat pasteurization, which has been more recently investigated (9–13).

Regarding microbial safety, both processes (LTLT and HTST) lead to appropriate inactivation of the vegetative forms of pathogens; however, these methods are completely ineffective against bacterial spores from exogenous contamination. During the last few years, two other human milk treatments have been developed: UV (14–16) and ultrasound (17, 18).

In terms of preserving the activity of human milk after these pasteurization treatments, the LTLT process leads to many components (with nutritional enzymatic and immune properties) with reduced activity.

Over the last 25 years, high hydrostatic pressure (HHP) processes have been developed in food processing to mainly induce microbial safety (19–22), which also consequently increases shelf life. Because HHP processes apply weaker energy than thermal ones, their main advantage is the preservation of the intrinsic properties of the treated medium. More recently, HHP processes have been extended to biological applications (23–30).

The first industrial developments of HHP processes were established in Japan (1985–1990). In this first approach (called a “conventional approach”), HHP processes were defined by only three main parameters: pressure (**P**), temperature (**T**), and duration of treatment (**t**). When HHP processes are managed by only these three parameters, high pressure values (450–600 MPa) must be applied to ensure high microbial safety (31), which has a negative consequence of inducing the modification of biological components or organoleptic properties of the handled product (32). If high temperatures (80–120°C) are not used to induce detrimental modifications to biological activity, these HPP processes are ineffective at spore inactivation (33).

## Definition of an HHP process applied to human milk

Different applications of this “conventional approach” to HHP treatment of human milk were tested with an emphasis on their ability to improve microbial safety. Viazis et al. (34) applied constant pressure (400 MPa) to human milk inoculated with different microorganisms [*Staphylococcus aureus* (ATCC 6538 and ATCC 25923)*, Streptococcus agalactiae* (ATCC 12927)*, Listeria monocytogenes* (ATCC 19115), and *Escherichia coli* (ATCC 25922)] to compare LTLT thermal pasteurization (Holder process) to high pressure treatment. The starting temperature was close to 21°C to reach a temperature of ~31°C due to adiabatic compression heating. Six- to eight-log reductions were observed in microbial populations during treatment. Unfortunately, this HHP treatment used a conventional approach and was ineffective against bacterial spores, particularly *Bacillus cereus* spores, which represent a microbial strain observed in the contamination of fresh milk, heat-treated milk and human milk (35, 36).

### Research aim

To establish an HHP process to inactivate both vegetative forms and bacterial spores contaminating human milk while preserving a substantial portion of the activity of milk components.

## Methods

Considering that high temperatures are rejected and that the pressure–temperature range required for spore inactivation would also lead to strong alterations of the biological activity of human milk components, an HHP process that could induce the germination of bacterial spores at lower pressure conditions (a moderate pressure value: *P* ≈ 350 MPa) was needed to preserve the biological activity of human milk as required for infant feeding.

Recently, a new approach to HHP processes was established by Demazeau et al. (37), and this approach accounts for parameters that characterize pressure delivery. Specifically, the compression rate (**VA**) or decompression rate (**VD**), application mode (**MA)** (continuous or cyclic) and latency time (**t**_l_) between each cycle were defined.

### Design

(i) To prove that this novel HHP was efficient for all pathogens with vegetative and spore forms, we performed a “challenge test.” To validate this novel approach to HPP processes for the decontamination of human milk, we inoculated sterilized human milk at a level of 6 log with two main strains of microorganisms: *S. aureus* (ATCC 6538), which is a gram-positive vegetative bacterium resistant to pressure inactivation (38), and bacterial spores of *B. cereus* (ATCC 14579), a sporulated bacterial form that can induce severe intestinal infections (39).
After applying various optimization tests, we defined the HHP experimental conditions based on 6 parameters that can inactivate all vegetative forms and bacterial spores (such as *B. cereus* spores).
The set of optimized process parameters was as follows:
Pressure = 350 MPa, temperature = 38°C, VA (application rate) = 1 MPa.s^−1^, MA (application mode) with n_a_ (number of cycles) = 4 cycles and t_a_ (duration of each cycle) = 5 min, and t_l_ (latency time with normal pressure between each cycle) = 5 min.
(ii) To demonstrate the conservation of bioactive components of human milk, we compared raw human milk with pasteurized and HHP treatments of the same sample. We measured the main biologic components of human milk, including lipase activity, lactoferrin, lysozyme, and IgA under either Holder pasteurization (62.5°C, 30 min) or novel high hydrostatic pressure.

### Setting

The high-hydrostatic pressure machine is located at HPbioTech, which is situated 10 km from the Human Milk Bank (HMB) of Bordeaux-Marmande.

We used human milk after consent from the mother. Raw human milk is pasteurized and stored at −18°C at HMB; when transferred to HPbioTech, it is stored at −80°C until analysis.

### Sample

The pasteurized human milk was used to set of optimized process parameters of HHP. Sterile human milk was inoculated with 5–6 log *S. aureus* or *B. cereus* and treated with the optimized set of HHP. This was termed the “Challenge test.”

The raw human milk was treated with the optimized set of HHP to measure the biologic products of human milk, such as lipase activity and immune proteins lactoferrin, lysozyme, IgAs.

After HHP treatment, the challenge was employed to identify conditions that allow for destroying vegetative and spore forms of bacteria and preserving lipase activity and immune bioactive proteins.

Compared to Holder pasteurization, which destroyed 0 spores (see Table 1), the HHP sample size was determined to be 6 log of *B. cereus* spores and 6 log of *S. aureus*. The value obtained after Holder pasteurization showed no destruction of *B. cereus*, but no *B. cereus* was found after HHP treatment. Thus, very few samples are needed (see Table 1). We measured the reproducibility of destroying 6 log *B. cereus* in 3 repeated HHP treatments.

**Table 1:** Inactivation Efficiency (IE) of *B. cereus* (ATCC 14579) (as spores) and *Staphylococcus aureus* (ATCC 6538) after the new High Hydrostatic Pressure (HHP) treatment.

	**N_i_**	**N_HHP_**	**IE**
**Microorganism (bacteria sporulated form)**^a^			
*B. cereus* control D11	4.9	−	−
*B. cereus* HHP8 D11 n°1	4.9	0	4.9
*B. cereus* HHP8 D11 n°2	4.9	0	4.9
*B. cereus* HHP8 D11 n°3	4.9	0	4.9
**Microorganism (vegetative form)**^b^			
*S. aureus* control D11	5.7	−	−
*S. aureus* HHP8 D11 n°1	5.7	0	5.7
*S. aureus* HHP8 D11 n°2	5.7	0	5.7
*S. aureus* HHP8 D11 n°3	5.7	0	5.7

a *Inactivation Efficiency (IE) of B. cereus (ATCC 14579) (as spores) after the new HHP treatment. The inactivation efficiency of the HHP process for human milk inoculated with Staphylococcus aureus (ATCC 6538) and the evaluation of its reproducibility (n°1, n°2, n°3) are given on Table 2. N_i_ and N_HHP_ are respectively the initial (before the HHP treatment) and final (after the HHP treatment) microbial contamination. IE corresponds to the Inactivation Efficiency of the HHP treatment*.

b *Inactivation Efficiency (IE) of Staphylococcus aureus (ATCC 6538) after the new HHP treatment. The inoculation rate was limited to 5.7 log the initial contamination of human milk accepted for a decontamination treatment (as LTLT as the present time) by Staphylococcus aureus being limited to 4 log due to the release of toxins (40)*.

The Holder treatment destroyed all lipase activity (activity = 0), whereas between 70 and 100% of residual activity was found with the HHP treatment (see Table **3**).

### Data analysis

We first verified the normality of the population and the homoscedasticity of variances. If verification was achieved, we used the Student *t* test to compare the two treatments (Holder vs. HHP); if not, we used the non-parametric test.

Two tailed *p* < 0.05 indicated significance.

### Ethical consideration

The milk used in this study was derived from the Human Milk Bank of Bordeaux-Marmande. Prior to donating milk, each mother signed a consent form indicating that any discarded milk could be used for research purposes. We therefore did not require approval for this study from the local Ethics Committee.

Moreover we can utilize human milk samples that cannot be used because the mother smokes or there are other contraindications to its donation.

## Measurement

### Protein and lipid analyses

#### Proteins

**Figure 1:**
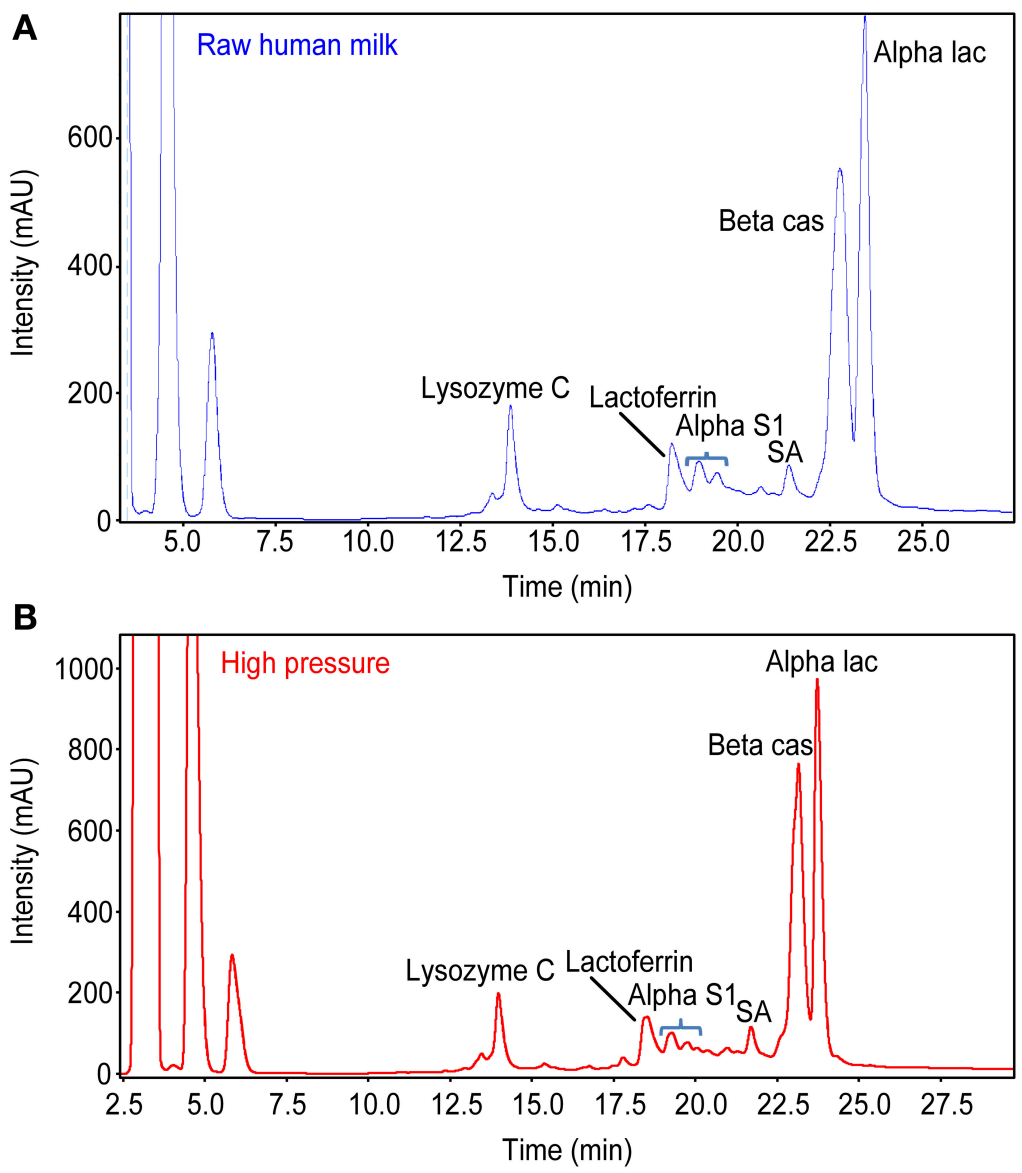
Comparison of proteins profile between **(A)** Raw Human Milk and **(B)** HHP Human Milk. There is strictly the same proteins profile of Raw HM and HHP HM.

Caseins and the main soluble proteins were analyzed qualitatively and quantitatively using RP-HPLC coupled with Electro Spray Ionization-Mass Spectrometry (LC-MS); 50 samples of the same batch of raw HM (50), LTLTHM (50) and HPPHM (50) (INRA Jouy en Josas, Dr. P. Martin) were used (Figure 1).

Bioactive, antimicrobial and immune proteins: lactoferrin, lysozyme and IgA, showing more or less broad ranges of functions, were analyzed using Enzyme Linked ImmunoSorbent Assays (ELISAs) based on the sandwich technique; the antibody directed against the protein to analyze is pre-coated on the surface of microtiter wells. A biotinylated detection antibody is then added to the wells to bind to the captured protein. Streptavidin-conjugated horseradish peroxidase (SA-HRP) is then added to catalyze a colorimetric reaction with the chromogenic substrate 3,3′,5,5′-tetramethylbenzidine. The colorimetric reaction produces a blue product, which turns yellow when the reaction is terminated by addition of dilute sulfuric acid. The absorbance of the yellow product at 450 nm is proportional to the amount of protein present in the sample. The protein concentrations in the test samples can then be quantified by interpolating their absorbance from the standard curve generated in parallel with the samples (41).

#### Lipids

Lipase activity (Institut Biochimie Nutrition ITERG, Dr. C. Vaysse–Dr. L. Couedelo). The lipase activity compared to the substrate was monitored by quantitative release of fatty acids and glycerol generated during the hydrolysis of TAG lipase. The hydrolysis reaction is automatically followed by pH measurements via neutralization of fatty acids liberated by the enzyme over time by a standard solution of sodium hydroxide (NaOH 25 mmol/L) at pH 8 ± 0.2.

The results are expressed as the in International Units as micromoles of fatty acids liberated per minute and per milliliter of breast milk.

**Figure 2:**
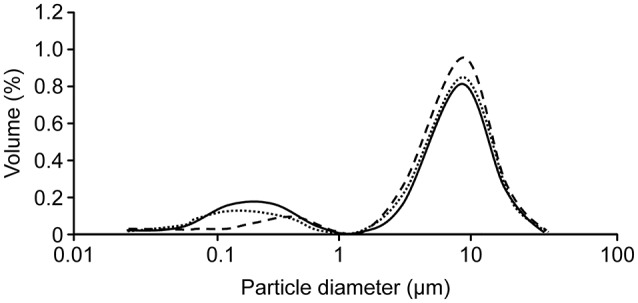
Distribution of the volume size of milk fat globules (MFGs): raw (**____**) pasteurized (- **- - -**) or high pressure (**………**). Evaluation of the size of MFGs showed that the population was bimodal he proportion of “small MFGs” was greater in raw milk and HHP-treated milk (d3.2 = 0.6 vs. 0.8 μm, respectively) compared to that in LTLT-pasteurized human milk (d3.2 = 3.1 μm). This result suggests that the size structure of raw breast milk is preserved by HHP treatment, whereas LTLT promotes coalescence and therefore increases the number of “large” MFGs.

We used the granulometry of lipid droplets of human milk: raw, pasteurized, and novel HHP of the same batch (Figure 2).

## Results

### Microbial spore and vegetative destruction

In these experimental conditions, total inactivation of the microbial contamination of human milk was possible in challenge tests with *S. aureus* and *B. cereus* spores.

The IE of the new HHP process for human milk inoculated with spores of *B. cereus* (ATCC 14579) and an evaluation of its reproducibility (n°1, n°2, and n°3) are provided in Table 1. N_i_ and N_HHP_ are, respectively, the initial (prior to HHP treatment) and final (after HHP treatment) microbial concentrations. IE is provided for HHP treatment

Tables 1, 2 provide the corresponding inactivation efficiency (IE) of each microorganism. The HHP experiment and microbial analysis were repeated 3 times (n°1, n°2, and n°3) for the same human milk samples, with parameters of New HHP-*P* = 350 MPa, *T* = 38°C, MA = 4 × 5 min, Tl = 5 min, VA = 1 MPa/s

**Table 2:** The effect of High Hydrostatic Pressure (HHP) on 10 samples of (1.5 × 106) colonies of *Bacillus cereus*; HHP destroyed all the colonies, i.e., 1.5 × 106 colonies with Inactivation Efficiency (IE) = 6.18 Log.

**Microorganism (spore form)**	**N (UFC/mL)**	**N (Log)**	**IE**
HM B.cer control	1.5 × 106	6.18	–
HM B.cer 1 HP	0	0	6.18
HM B.cer 2 HP	0	0	6.18
HM B.cer 3 HP	0	0	6.18
HM B.cer 4 HP	0	0	6.18
HM B.cer 5 HP	0	0	6.18
HM B.cer 6 HP	0	0	6.18
HM B.cer 7 HP	0	0	6.18
HM B.cer 8 HP	0	0	6.18
HM B.cer 9 HP	0	0	6.18
HM B.cer 10 HP	0	0	6.18

*Tables 1, 2 provide the corresponding IE of each microorganism. The HHP experiment and microbial analysis were repeated 3 times (n°1, n°2, and n°3) for the same human milk samples, with parameters of New HHP-P = 350 MPa, T = 38°C, MA = 4 × 5 min, Tl = 5 min, VA = 1 MPa/s*.

The effect of HHP on 10 samples (1.5^E^+06), whereby colonies of B. Cereus were all destroyed

i.e., 1.5E+06 Colonies Inactivation Efficiency (IE) = 6.18 Log

In addition, this HHP process was evaluated for the inactivation of cytomegalovirus (CMV). This virus was selected due to its risk of human milk contamination and risk of postnatal infection (40, 42–44).

Different attempts were made using a suspension of cytomegalovirus with an initial viral particle concentration of up to 7 log. After application of the HHP treatment, which was characterized by the optimized process parameters used for the inactivation study of either *S. aureus* or bacterial spores of *B. cereus*, total CMV inactivation was observed.

### Activity retention of the main constituents of human milk

As a first evaluation, three main types of human milk components were selected:

- A component with enzymatic properties (lipase),
- Components with antimicrobial properties (lysozyme and lactoferrin) or with immunological properties (IgA), and
- Components with nutritional properties.

#### Impact of the HHP process on lipase activity

An evaluation of lipase activity was important to compare the biological effects of this HPP process with those of thermal LTLT treatment (induction of total inactivation). The experimental conditions of HHP treatment were the same as those used for microbial decontamination (*P* = 350 MPa, *T* = 38°C, VA = 1 MPa.s^−1^, MA = 4 cycles of 5 min and t_l_ ± 5 min).

Residual activities of the lipase enzyme in three samples (raw milk, LTLT-pasteurized milk and HHP-treated milk) and in three replicates (n°1, n°2, and n°3) for HHP treatment are provided in Table 3. Due to the variability of lipase activity in human milk, three different human milk samples (A, B, and C) were used. The lipase activity was completely destroyed (0% lipase activity) by LTLT, whereas lipase activity after the new HHP remained similar to its original value (Raw Human Milk) between 78.6 and 100% with a mean of 87.8% (Wilcoxon test *p* = 0.25) (Table 3).

The residual activity of the lipase enzyme after HHP processing of human milk was between 80 and 85% of the initial value of human milk treated at 38°C.

**Table 3:** Lipase activity of raw human milk samples and milk samples after thermal “Low-Temperature, Long-Time” (LTLT) pasteurization and High Hydrostatic Pressure (HHP) processing.

**Sample**	**Lipase activity**	**% Lipase activity**
Raw milk A	0.57	100
Raw milk B	0.71	100
Raw milk C	0.70	100
**LTLT-PASTEURIZED MILK**		
Pasteurized A	0.00	0.00
Pasteurized B	0.00	0.00
Pasteurized C	0.00	0.00
**HHP-TREATED MILK**		
HHP A n°1	0.57	100
HHP A n°2	0.53	92.9
HHP A n°3	0.53	92.9
HHP B n°1	0.68	95.8
HHP B n°2	0.56	79
HHP B n°3	0.58	81.7
HHP C n°1	0.59	84.3
HHP C n°2	0.55	78.6
HHP C n°3	0.60	85.7

*(A, B, and C correspond to 3 different human milk samples, and n°1, n°2, and n°3 correspond to HHP treatment reproducibility assays. Lipase activity of raw human milk samples and milk samples after thermal LTLT pasteurization and HHP processing (A, B, and C correspond to 3 different human milk samples, and n°1, n°2, and n°3 correspond to HHP treatment reproducibility assays) The lipase activity was completely destroyed (0% lipase activity) by LTLT, whereas lipase activity after the new HHP remained similar to its original value (Raw Human Milk) between 78.6 and 100% with a mean of 87.8% (Wilcoxon test p = 0.25)*.

A comparison with the residual activity from the conventional HHP process [reproducing Viazis's et al. (34) HHP treatment] resulted in a residual lipase activity of close to 75%.

#### Impact of the HHP process on the activity of different components with antibacterial or immune properties

The activity of biological components (lysozyme, lactoferrin, α-lactalbumin, and IgA) was also evaluated before and after HHP processing of human milk using the same set of experimental parameters (Table **5**; Figure 1).

Considering the differences between our HHP process and those described in the literature for human milk, the following remarks can be made.

#### Impact of the HHP process on human milk components with nutritional properties milk fat globule (MFG) granulometry in raw, LTLT-pasteurized, and HHP-treated human milk using this novel HHP process

Evaluation of the size of MFGs showed that the population was bimodal with an approximately equivalent average diameter (d4.3) for all types of milk (raw milk: 5.5 μm; LTLT: 5.6 μm; and HHP: 5.4 μm). In addition, the proportion of “small MFGs” was greater in raw milk and HHP-treated milk (d3.2 = 0.6 vs. 0.8 μm, respectively) compared to that in LTLT-pasteurized human milk (d3.2 = 3.1 μm). This result suggests that the size structure of raw breast milk is preserved by HHP treatment, whereas LTLT promotes coalescence and therefore increases the number of “large” MFGs. In addition, the total fat content was similar regardless of the performed treatment (raw, LTLT and HHP: 34.0, 34.1, and 32.3 mg/mL milk, respectively) and of the fatty acid profile of the milk.

## Discussion

For the first time, this new HHP process for the microbial safety of human milk can irreversibly inactivate both the vegetative forms of microorganisms, such as gram-positive bacteria including *S. aureus*, and **bacterial spores**, such as those of the contaminant *B. cereus*, while preserving at least 80% of the biological activity of the main components. Previously reported works involving HHP treatments were based on so-called “conventional” approaches in which the applied pressure was not controlled (15). Table 4 provides average values of the resulting microbial safety using three processes (LTLT, HTST and this new HHP) on human milk with *S. aureus* (gram-positive bacterium) and *B. cereus* (sporulated bacterium) as contamination references. Consequently, inactivation of bacterial spores, such as those of *B. cereus*, was not possible with a technique other than the new HHP.

**Table 4:** Average values of the resulting microbial safety using *Staphylococcus aureus* (Gram-Positive Bacterium) and *Bacillus cereus* (Sporulated Bacterium) as contamination references.

	**Inactivation efficiency (IE)**		
	**LTLT^a^**	**HTST^b^**	**New HHP^c^**
*S. aureus*	≈4	≈4	≈6
*B. cereus* spores	No effect	No effect	≈5

a *Higher contamination accepted for the Holder treatment (10^4^ CFU /mL) due to toxin production by S. aureus*.

b *Giribaldi et al. (45)*.

c *This paper*.

The retention rates of the biological activity for different components with specific properties [BSSL lipase (with enzymatic properties), lysozyme and lactoferrin (characterized by antimicrobial properties), and IgA (with immunological properties)] are summarized in Table 5.

**Table 5:** Average retention rates of biological activity for different components with specific properties after “Low-temperature, long-time” (LTLT), “High-temperature, short-time” (HTST) and the new “High Hydrostatic Pressure” (HHP) treatments were applied to human milk.

	**Retention rates (% vs. the raw milk)**		
	**LTLT**	**HTST**	**New HHP**
BSSL (lipase)	0–10 (45)	26 (45)	80–85 (this paper)
Lysozyme	52.3	48.8 (45)	> 95 (this paper)
Lactoferrin	≈ 20 (12)	30–40 (12)	93–97 (this paper)
IgA	46.3	78.9 (45)	64 (this paper)

Comparisons with the HTST process suggest that the retention rates of the biological activity of human milk components vary widely (particularly for BSSL) by author (9–13). In a recent paper by Giribaldi et al. (45), two aspects of the impact of the HTST process were evaluated: (i) microbial inactivation but not destruction of *B. cereus* and microbial spores and (ii) retention of the biological activity of human milk components using a specific HTST device for human milk pasteurization. Residual lysozyme activity was between 95% and 100% after application of our HHP process. Our value agrees with that reported by Viazis et al. (46) (96%) following HHP treatment of human milk at 400 MPa and 20°C. Viazis's et al. (34) HHP treatment resulted in a residual lipase activity of close to 75%.

Mayayo et al. (47) found that treatment at 300, 400, 500, and 600 MPa for 15 min and *T* = 20°C using the “conventional approach” to HHP processes denatured 9, 23, 34, and 48% of lactoferrin, respectively. In our approach, the retention rate of lactoferrin was over 93% (denaturation was below 7%) despite using a temperature of 38°C to limit the germination of *B. cereus* spores.

The residual activity of IgA was comparable to that obtained by Delgado et al. (48) (47.5% at 300 MPa and 50°C). In an early paper, Viazis et al. (46) found that high-pressure processing of human milk using the “conventional approach” at 400 MPa for 30, 60, 90, and 120 min and at a treatment temperature close to 31°C resulted in 85.6, 87.1, 80.6, and 75.4% retention, respectively. Permanyer et al. (49) claimed that after a treatment at 400 MPa for 5 min at 12°C, 100% of IgA activity was maintained, whereas IgA retention was 87.9 and 69.3% at higher pressure conditions (500 and 600 MPa, respectively). Contador et al. (50) evaluated the retention activity of IgA after high-pressure treatment at different pressures (400 and 600 MPa) and different treatment durations (3 and 6 min) with an initial temperature of 10°C at 400 MPa for 6 min; the retention of IgA activity was close to 90%.

Comparisons of these research studies suggest that IgA activity mainly depends on both the pressure and temperature of high-pressure treatment.

The retention rates of the biological activity for different components with specific properties [BSSL lipase (with enzymatic properties), lysozyme and lactoferrin (characterized by antimicrobial properties), and IgA (with immunological properties)] are summarized in Table 5.

## Limitations

The new HHP process requires 90 min to treat human milk vs. 60 min for the Holder method; however, the cost of the HHP device is more than is a conventional pasteurizer. However, the new HHP saves up to 10% of material contaminated by *B. cereus*. For example, the Bordeaux Human Milk Bank collects 11,000 liters of human milk per year; 10% of this amount (or 1,100 liters) is contaminated with *B. cereus* and therefore must be discarded (51). This represents 165000€ /year, lost with the conventional pasteurizer per year, which would not be rejected with the new HHP.

## Conclusion

This new HHP process is promising for implementation in human milk banks based on a comparison of three processes for the microbial safety and retention of the biological activity of different milk components. This approach is the first process that can inactivate bacterial spores, such as those of *B. cereus*; this point is important due to the risks of bacterial spores to preterm or young infants (52).

## Author contributions

GD and AP processed the human milk samples, and the results of bacteriological analyses were verified in double-blind experiments performed by PL of CHU. GD worked with AP to write the first version of the manuscript; unfortunately, GD is now deceased. PL performed a double-blind bacteriological study on the HHP samples from GD at the CHU. LC and PM performed the experiments, analyzed the data and wrote their part of manuscript and participated in revising the manuscript. Technis and results of Bacteriology was revised by PL and AP. The technics and results of Lipids by LC. The technics and results of proteins by PM. CB wrote the manuscript.

### Conflict of interest statement

This new high-pressure hydrostatic process was developed by GD (Pr. Emeritus at the Science University Bordeaux) who created a start-up called HPbiotech. He cooperated with the Centre Hospital University of Bordeaux, and in particular with CB, to coordinate a study of a new high hydrostatic pressure (HHP) process capable of destroying all vegetative and spore forms of pathogens. His research was protected by a patent (HPbiotech-CHU Bordeaux) prior to any publication. This process was not marketed, and this study was financed by a grant of 150000€ from the Conseil Regional d'Aquitaine. AP is a paid employee of HPbiotech. CB performs industrial and public research for Nestle, but these industrial grants do not interfere with the research described herein concerning the safety of donated human milk. All analyses were funded by the previously mentioned grant from the Conseil Regional d'Aquitaine. The remaining authors declare that the research was conducted in the absence of any commercial or financial relationships that could be construed as a potential conflict of interest.
